# Recombinant zoster vaccine immunization rate in patients on Janus kinase inhibitors for rheumatologic and dermatologic conditions

**DOI:** 10.1093/rap/rkag071

**Published:** 2026-06-11

**Authors:** Jessica B Michaud, Ruta Brazauskas, Sarah Wigfield, Shelby Shepard, Teresa Mysliwiec, Kylie Perez, Shikha Singla

**Affiliations:** Rheumatology, Froedtert & the Medical College of Wisconsin, Milwaukee, WI, USA; Rheumatology, Froedtert & the Medical College of Wisconsin, Milwaukee, WI, USA; Rheumatology, Froedtert & the Medical College of Wisconsin, Milwaukee, WI, USA; Pharmacy, University of Iowa Health Care, Iowa City, IA, USA; Rheumatology, Froedtert & the Medical College of Wisconsin, Milwaukee, WI, USA; Rheumatology, Froedtert & the Medical College of Wisconsin, Milwaukee, WI, USA; Rheumatology, Froedtert & the Medical College of Wisconsin, Milwaukee, WI, USA

**Keywords:** Janus kinase inhibitors, herpes zoster, vaccination, immunization, rheumatologic, dermatologic, pharmacist

## Abstract

**Objectives:**

Patients who receive Janus kinase (JAK) inhibitors are at an increased risk of varicella zoster virus reactivation. The primary objective of this study was to evaluate the vaccination rates of recombinant zoster vaccine (RZV) for patients taking a JAK inhibitor for dermatologic or rheumatologic conditions.

**Methods:**

A facility-based cross-sectional study design was employed to select patients who were ≥18 years of age and were prescribed a JAK inhibitor for at least 6 months by a rheumatologist or dermatologist for any rheumatologic or dermatologic condition. Data analysis was undertaken using descriptive methods.

**Results:**

There were 102 patients included in this study, with most patients being female (77.5%), ≥50 years of age (71.6%), White (77.5%) and with RA (76.5%). The proportion of patients who received at least one RZV (at any time in relation to JAK inhibitor initiation) was 27.5% (28/102). Only three patients received at least one dose of RZV within 30 days before or after initiation of a JAK inhibitor.

**Conclusion:**

Many patients receiving JAK inhibitors are not vaccinated with RZV, and of those who are vaccinated, the timing of vaccination rarely coincided with JAK inhibitor initiation and thus did not appear to be the motivation for vaccination. These data will be used to optimize vaccination workflow within ambulatory clinics to mitigate herpes zoster risk in this population.

Key messages

- For patients on a JAK inhibitor, there is an increased risk of herpes zoster reactivation.
- The majority of patients on a JAK inhibitor for a rheumatologic or dermatologic condition did not receive an RZV.
- These findings may help to promote discussion among providers and patients to improve RZV rates among patients on a JAK inhibitor for rheumatologic or dermatologic conditions.

## Introduction

Janus kinase (JAK) inhibitors are a class of small molecules utilized for various immune-mediated diseases, including but not limited to RA, PsA, AS, non-radiographic axial SpA, JIA, IBD, atopic dermatitis, alopecia areata, plaque psoriasis [^1–3^] and myeloproliferative diseases. [4] Tofacitinib, baricitinib, upadacitinib, abrocitinib, ritlecitinib and deuruxolitinib are the oral JAK inhibitors approved for rheumatologic and dermatologic conditions, the latter two being more recently approved by US Food and Drug Administration (FDA). While these medications have been proven effective, therapies targeting JAK complexes are associated with a higher risk of herpes zoster infection than other immunomodulating therapies. [5] The best comparison was a head-to-head safety trial in patients >50 years of age with RA and cardiovascular risk, showing patients taking tofacitinib 5 mg two times per day had a relative risk (RR) of 3.28 (95% CI 2.44, 4.41) compared with a TNF inhibitor. [6] JAK inhibitors are also implicated in varicella zoster reactivation in a number of clinical trials. [7–10]

The recombinant zoster vaccine (RZV) known as Shingrix^®^ is a recombinant, adjuvanted zoster vaccine that was approved by FDA in 2017 to prevent herpes zoster (shingles) for patients ≥50 years of age.^11^ RZV is a two-dose vaccine series and has been shown to be very effective in the prevention of herpes zoster. Based on two phase III clinical trials, ZOE-50 and ZOE-70, the RZV series was 97.2% effective in preventing herpes zoster in patients ≥50 years of age and 91.3% in patients ≥70 years of age.^12^^,^^13^

In July 2021, FDA expanded the RZV indication to include immunocompromised patients ≥18 years of age.^14^ In October 2021, the Centers for Disease Control and Prevention (CDC) also expanded their recommendation to this population based on five studies evaluating vaccinations in post-autologous haematopoietic stem cell transplant (HSCT) recipients, HIV-infected adults, patients with haematologic malignancies, and patients ≥50 years of age with potential immune-mediated diseases. Efficacy rates, based on herpes zoster infection, ranged from 68.2% in patients post-autologous HSCT to 90.5% in patients with potential immune-mediated diseases.^14^

In 2019, the National Psoriasis Foundation issued a consensus recommendation that stated, ‘Recombinant zoster vaccine is recommended for all psoriasis and PsA patients >50 years old and patients <50 years old on tofacitinib, systemic steroids, or combination systemic treatment’.^15^ Similarly, the 2022 ACR Guideline for Vaccinations in Patients with Rheumatic and Musculoskeletal Diseases (RMD) recommended, ‘For patients with RMD age >18 years who are taking immunosuppressive medication, administering the recombinant VZV vaccine is strongly recommended’.^16^ The most recent EULAR guideline also notes the importance of herpes zoster vaccination in autoimmune inflammatory rheumatic diseases (AIIRDs), stating the live-attenuated herpes zoster vaccine ‘may be considered in high-risk patients with AIIRD’; however, as the guidelines were from 2019, and the non-live vaccine was licensed in Europe in March 2018, there are no specific recommendations regarding RZV.^17^

While herpes zoster vaccination is explicitly recommended in certain rheumatology and dermatology populations, and there are studies on the immunogenicity and safety of RZV in similar populations and on herpes zoster infection and risk factors, there is a gap in knowledge about the actual rate of vaccination in the rheumatology and dermatology populations, especially those taking JAK inhibitors, which, at the time of study development, had no published papers. The purpose of this study is to assess the rate of RZV vaccination for patients prescribed JAK inhibitors by rheumatologists or dermatologists at a single centre in North America.

## Methods

We conducted a single-centre, cross-sectional study to evaluate RZV uptake. Patients were included if they were ≥18 years of age and had been prescribed tofacitinib, baricitinib, upadacitinib or abrocitinib for ≥6 months by a rheumatologist or dermatologist for a rheumatologic or dermatologic condition. The notable exclusion criterion was a contraindication to RZV. An electronic health record report was generated in May 2023 to identify all patients prescribed a JAK inhibitor by the rheumatology or dermatology departments at the time of report generation. The sample size was determined by the number of eligible patients at a single centre and a single time point. Manual chart review was performed to confirm eligibility and determine vaccination status using the medical record and the state immunization registry database. The study was approved by the Medical College of Wisconsin Institutional Review Board (PRO00047709). The requirement for informed consent was waived because the study consisted solely of a retrospective chart review of existing data, involved no direct interaction with participants and presented no more than minimal risk to subjects. This study aligns with the required elements of the Strengthening the Reporting of Observational Studies in Epidemiology checklist for cross-sectional studies.

The primary outcome was the proportion of patients who received one or more doses of RZV. Secondary outcomes included the proportion of patients ages 18–49 years who received one or more doses compared with patients ≥50 years of age and the proportion of patients who were fully vaccinated. Among patients vaccinated after JAK inhibitor initiation, secondary outcomes included the proportions receiving one or more doses, exactly one dose or exactly two doses; time to the first dose and, when applicable, time to the second dose after JAK inhibitor initiation; the proportion receiving one or more doses by age group (18–49 *vs* ≥50 years); and the proportion of patients seen by an ambulatory clinic pharmacist (defined as a dermatology or rheumatology clinic pharmacist encounter at the time of education/prescribing or within 1 month after JAK inhibitor initiation). Among patients vaccinated before JAK inhibitor initiation, secondary outcomes included the proportions partially vaccinated (one dose) and fully vaccinated (two doses) at baseline. Additional variables collected included documented reasons for non-receipt of RZV, the proportion of patients recommended to receive RZV after JAK inhibitor initiation and the presence of other herpes zoster risk factors (i.e. primary immunodeficiency, treatment with immunosuppressive drugs including prednisone and conventional synthetic DMARDs, HIV, malignancies, radiation, chronic kidney disease and asplenia). JAK inhibitor initiation was defined as the date of first prescription entered by the rheumatologist or dermatologist.

Data were summarized using descriptive statistics. Counts and proportions have been used to summarize categorical variables and medians and ranges were used for continuous variables.

## Results

A total of 142 patients were receiving a JAK inhibitor for a rheumatologic or dermatologic condition at the time of screening; 102 (71.8%) met the inclusion criteria. Most patients were ≥50 years of age (71.6%), female (77.5%) and White (77.5%). The most common indication for JAK inhibitor therapy was RA (76.5%), and while most patients did not have another autoimmune condition (87.3%), of those who did, Sjögren’s disease was the most common. Table 1 presents the baseline characteristics of the study cohort. In regard to herpes zoster risk, most patients were not prescribed prednisone at the time of report generation (90.2%) and did not have another immunocompromising condition (62.7%).

**Table 1:** Demographics (*N* = 102).

Characteristics	*n* (%)
Gender	
Male	23 (22.5)
Female	79 (77.5)
Age (years)	
18–49	29 (28.4)
≥50	73 (71.6)
Race	
White	79 (77.5)
Black	11 (10.8)
Asian	3 (2.9)
Native/Alaskan America	3 (2.9)
Other	6 (5.9)
JAK inhibitor	
Tofacitinib	64 (62.7)
Baricitinib	1 (1.0)
Upadacitinib	37 (36.3)
Abrocitinib	0 (0)
Indication for JAK inhibitor	
RA	78 (76.5)
PsA	7 (6.9)
Atopic dermatitis	4 (3.9)
Plaque psoriasis	3 (2.9)
Other	10 (9.8)
History of herpes zoster	
No	98 (96.1)
Yes	4 (3.9)
Current prednisone prescription	
No	92 (90.2)
Yes	10 (9.8)
5 mg/day	6
20 mg/day	2
30 mg/day	1
Missing	1
Other autoimmune disease	
No	89 (87)
Yes	13 (13)
Other immunocompromising condition	
No	64 (62.7)
Yes	38 (37.3)

**Figure 1:**
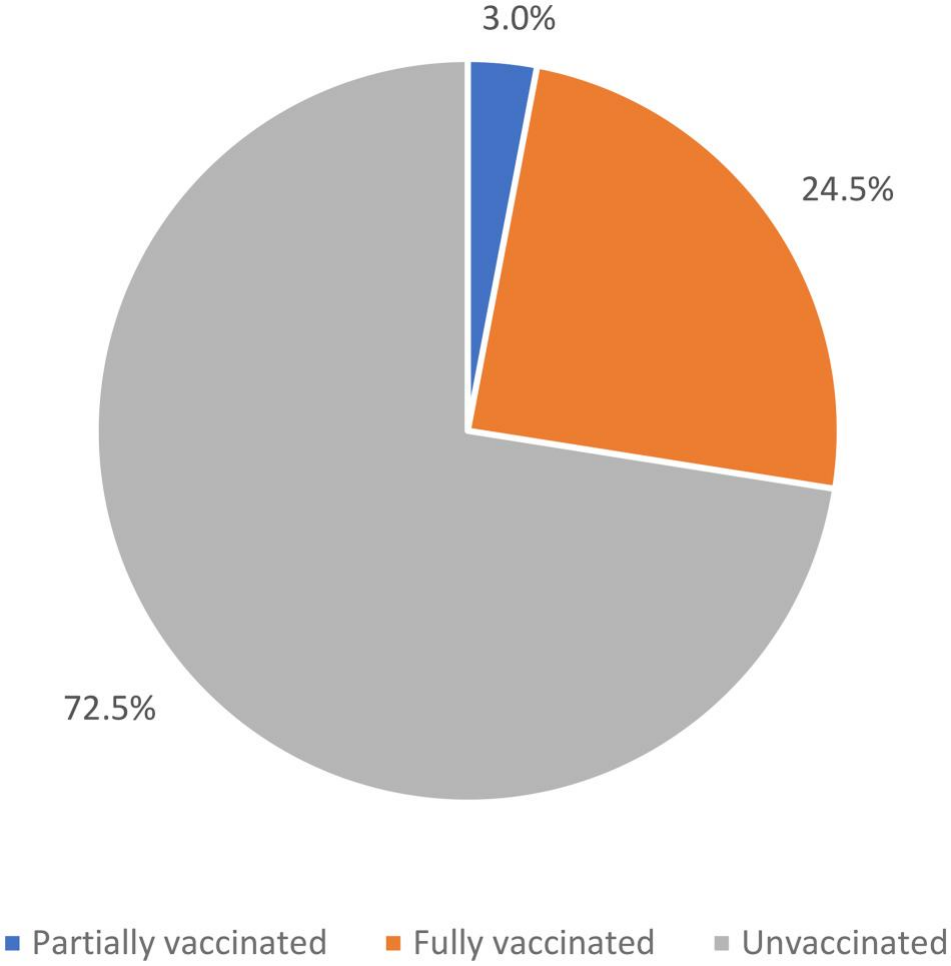
Percentage of patients that were partially (one dose) or fully (two doses) vaccinated or not vaccinated

**Figure 2:**
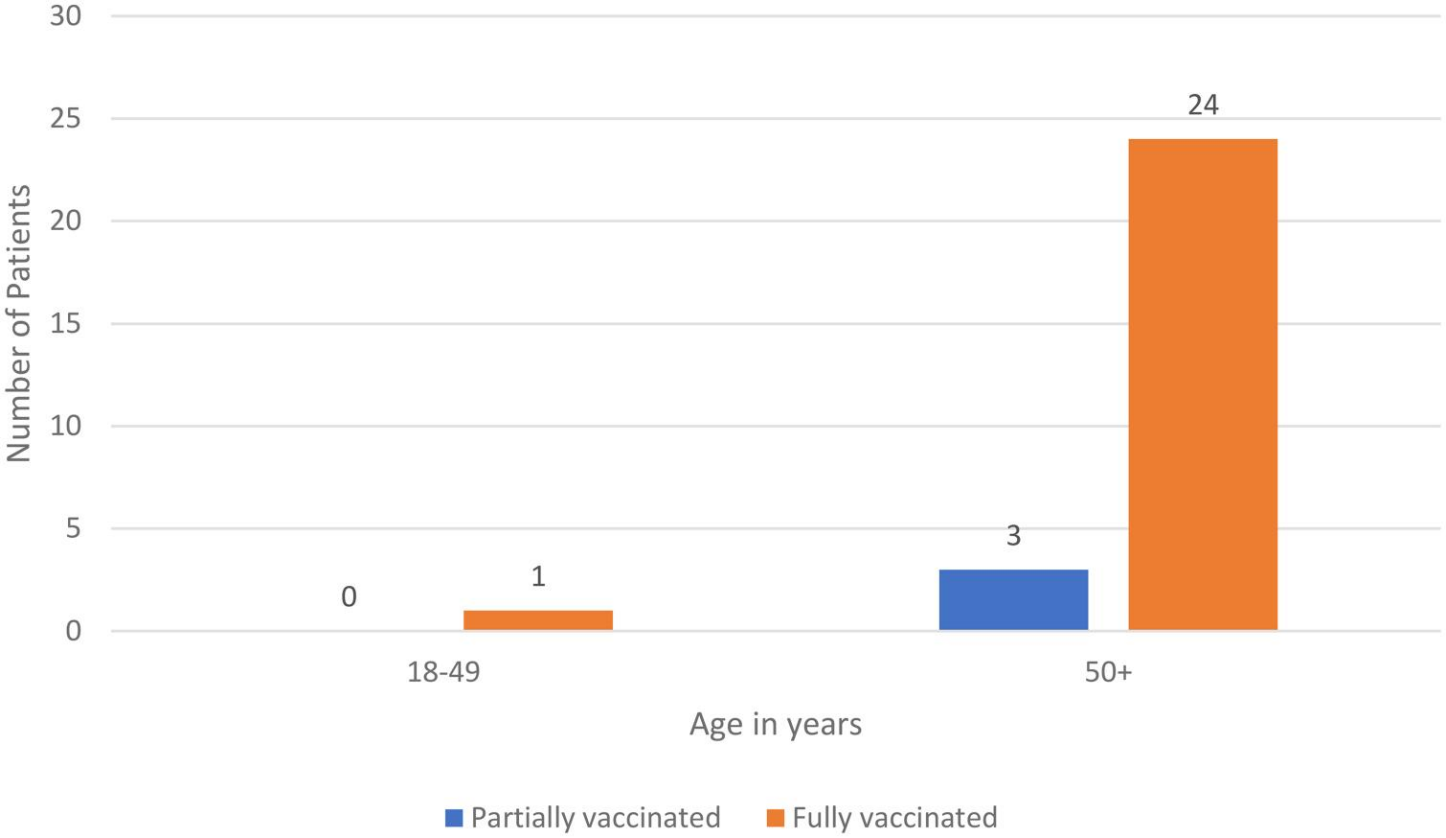
Number of patients partially (one dose) or fully (two doses) vaccinated based on age 18–49 years or ≥50 years

The proportion of patients who received one or more doses of RZV was 27.5% (28/102). Seventy-four patients (72.5%) did not receive RZV, 25 (24.5%) received two doses and 3 (3.0%) received one dose (Fig. 1). Only one patient <50 years of age received RZV, and that patient was fully vaccinated. Twenty-four patients (≥50 years of age) received two doses of vaccine (Fig. 2).

Among the latter, the median time to complete the series of two RZV doses was 504 days before JAK inhibitor treatment initiation. Eleven patients initiated RZV after starting JAK inhibitor therapy, with the median time of the first dose being 504 days; 8 of these 11 patients completed RZV with the second dose received at a median time of 998 days after JAK inhibitor initiation. Three patients received RZV within 30 days of JAK inhibitor initiation: one on the initiation date, one 6 days before and one 11 days before; these patients received the second dose at 62, 29 and 52 days after starting JAK inhibitor, respectively.

**Figure 3:**
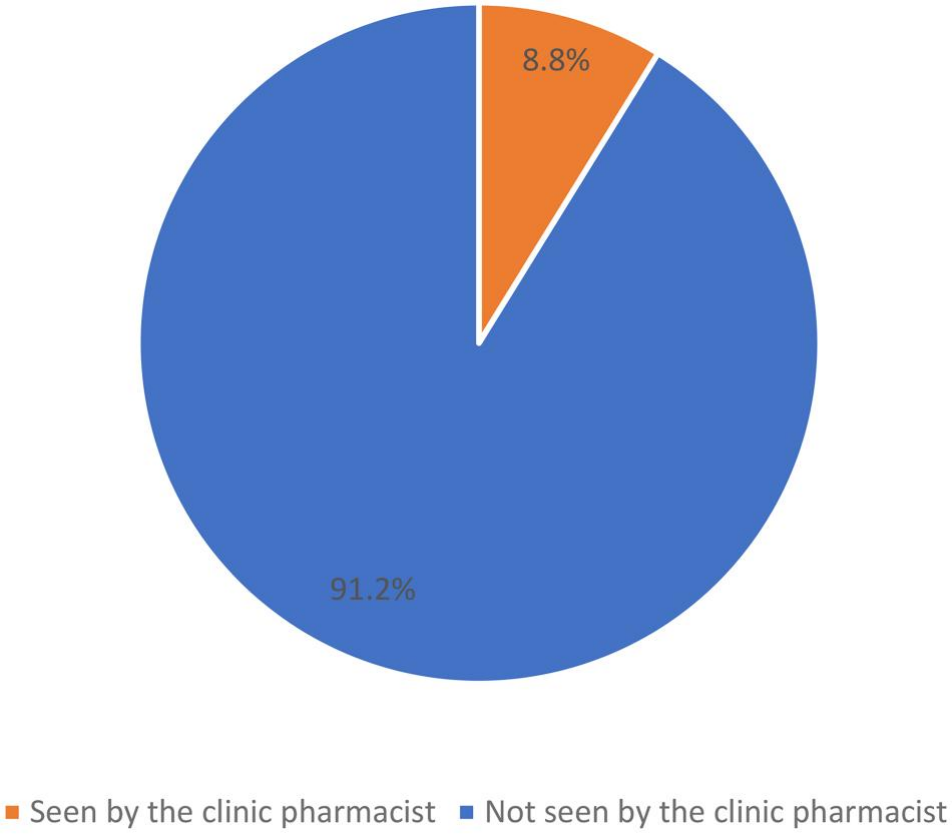
Percentage of patients seen by the clinic pharmacist during the time of JAK inhibitor initiation

**Figure 4:**
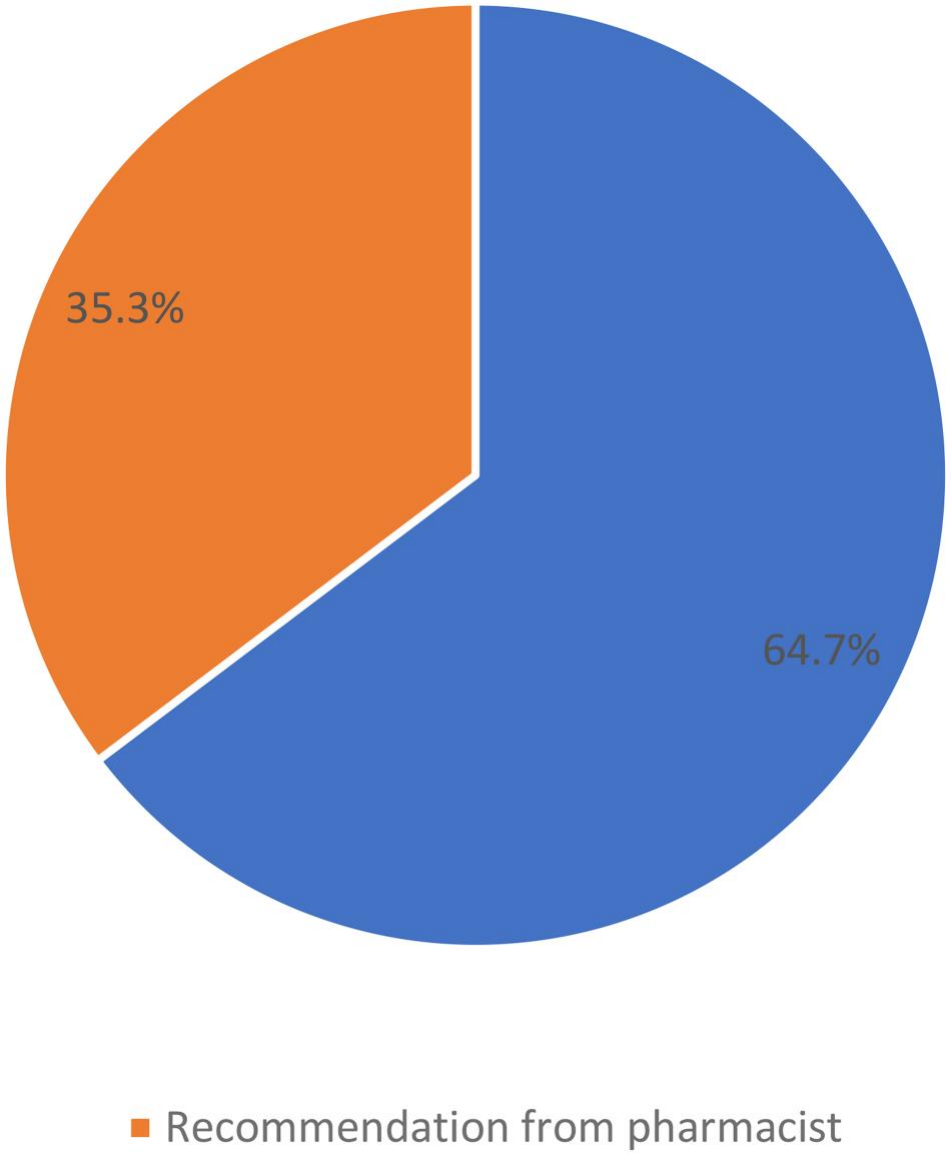
Percentage of recommendations for RZV provided by a pharmacist

A clinic pharmacist saw nine patients (8.8%) around the time of JAK inhibitor initiation (Fig. 3) and recommended RZV to half of those who were previously unvaccinated (four of eight). Overall, 34 patients (33.3%) received an RZV recommendation, including 12 (35.3%) recommendations made by pharmacists (Fig. 4). Among the 74 unvaccinated patients, only 7 had a reason documented for not receiving the vaccine, which included forgetting (*n* = 1), refusing (*n* = 4), indicating a previous incidence of shingles years prior (*n* = 1) or cost concerns (*n* = 1).

## Discussion

Patients taking immunocompromising medications, especially those on a JAK inhibitor, are at increased risk of varicella zoster reactivation. RZV has been proven to be effective and safe, not only in the general population, but also in patients on JAK inhibitors. The present study shows that the majority of patients had not received RZV, but those who did were nearly all ≥50 years of age. Only 1 of 29 patients between 18 and 49 years old received RZV. Patients who were vaccinated were nearly always fully vaccinated. The timing of vaccination in relation to the date of JAK inhibitor initiation suggests that the motivation for vaccination did not relate to the fact that a JAK inhibitor was being started, except for three patients, who were vaccinated on or just before starting the JAK inhibitor. A small percentage of patients received a documented RZV recommendation; however, a disproportionately high number of recommendations came from ambulatory clinical pharmacists. Most patients had no documented reason for not receiving RZV.

Herpes zoster vaccination rates in patients with immune-mediated inflammatory disease had been shown to be low (13%), and lower than other vaccines (influenza 69%, pneumococcal 35%), as reported in a cross-sectional study in a rheumatology clinic.^18^ Conducted before the development of our study, and similar to our research question, this study did not address specific immunosuppressant therapy as part of its methods with respect to inclusion criteria or results.

Since the time of the development of this study, there have been a few additional articles published that address a question similar to our study. One article, Hren *et al.*,^19^ showed a vaccination rate during the years 2003–2023 of 6.76% for herpes zoster in a dermatologic population prescribed a biologic or JAK inhibitor.

A second article, Curtis *et al.*,^20^ was a very large US database study during the years 2017–2023 in patients with inflammatory arthritis taking a variety of immunosuppressants. The results showed a vaccination rate of 21.6% for the first vaccine dose, and of those receiving a first vaccine dose, 73.2% received a second dose. Interestingly, they showed an increased risk of venous thromboembolism events in the first 60 and 90 days after herpes zoster infection, supporting the importance of improving vaccination rates. The authors also collected data on herpes zoster infection and calculated a crude vaccine effectiveness rate of 50%, lower than that found in studies of older immunocompetent patients. This lower effectiveness rate is supported by immunogenicity studies such as one by Sieiro Santos *et al.*,^21^ which showed lower seroconversion in patients treated with JAK inhibitors (74%) compared with patients treated with TNF inhibitors and/or methotrexate (91%) and control patients (100%, *P* < 0.001). An earlier small real-world study in patients with immune-mediated inflammatory disease on JAK inhibitors found similar results, with only 40% of patients achieving a quadrupling of varicella zoster antibodies.^22^ These data may highlight the importance of vaccine timing, ideally before active inflammatory disease and before initiation of immunosuppression, including JAK inhibitors.

A third article, Wong *et al.*,^23^ another large database study, found only ‘2.4% (*n *= 367/15 460) received HZ vaccination (Zostavax or Shingrix) within 6 months of b/tsDMARD commencement. There was only a small difference in HZ vaccination coverage in those commencing a Janus kinase inhibitor compared with a bDMARD (3.3% versus 2.1%, respectively, *P *< 0.001)’. This Australian rheumatologic study population was much larger than the current rheumatology/dermatology study population, but was collected during the years 2019–2021 and (for herpes zoster) included patients ≥50 years of age, before the CDC (and the Australian National Immunisation Programme) changed their recommendation for immunosuppressed patients not meeting minimal age requirements.

These data show work needs to be done to improve vaccination rates. While not documented well in the study population, barriers to vaccination may include busy clinics, concern for disease flare or other side effects, prior authorization requirement or lack of insurance coverage for RZV for patients <50 years of age, cost of vaccine, lack of coordination between clinic and pharmacy, lack of patient interest and lack of clarity regarding the best location (clinic *vs* pharmacy) for patients to receive RZV based on their insurance. A literature review by Neussser *et al.*^24^ showed that fear of adverse events is the most common barrier that prevents patients with rheumatic diseases from getting vaccinated. A survey of rheumatologists practicing in Ireland showed that only 5% of 44 respondents believed that the rheumatology clinic was the appropriate setting to administer vaccinations.^25^ About one-third (34%) of rheumatologists did not perform vaccination screening prior to initiating anti-TNF therapy. Twenty-one respondents (48%) favoured a primary care physician–led approach to manage immunization.

Jiang *et al.*^26^ conducted a cross-sectional survey that showed that the willingness of patients to get a vaccination may increase if recommended by physicians. Other possible tactics could involve the ambulatory care clinical pharmacist. A randomized study of an intervention that included both a communication to patients and a pharmacist review and prescription reported a statistically significant 2.7- to 2.9-fold increase in zoster vaccine live vaccination rates in intervention recipients compared with controls.^27^ A retrospective study to determine influencing factors on RZV series completion rates found that rates were considerably higher in the pharmacy claims subset *vs* the medical claims subset at 6 months (73% *vs* 49%), suggesting that community pharmacists improve vaccination rates.^28^ These results are similar to a study by Dauz *et al.*,^29^ who observed that pharmacist-driven immunization screening led to a statistically significant increase (1.9–20.2%; *P* < 0.05) in pneumococcal vaccination rate. Another study of 214 patients found that 39.2% (*P* = 0.001) and 45.8% (*P* = 0.01) of patients consented to receive influenza and pneumococcal vaccinations, respectively, after being counselled by pharmacists.^30^

At the institution where this study was completed, the pharmacist embedded within the rheumatology and dermatology clinics is informed when patients are newly starting JAK inhibitors and thus could contact patients at that time to provide education surrounding the importance of vaccination while on an immunomodulating medication and make the vaccine recommendation. The pharmacists are available in clinic to answer real-time vaccine questions, e.g. related to insurance or location of vaccination, and could directly coordinate with community pharmacists and retail pharmacies.

Strengths of the study include its novel study population of only patients taking JAK inhibitors for either a rheumatologic or dermatologic condition. The study was conducted 2 years after the FDA and CDC changes, allowing enough time for adoption of the changes while assessing vaccination rates based on the most updated recommendations.

The study has several limitations. First, the medical record may not include all vaccines received; e.g. vaccines administered outside the state will not always be recorded in the state immunization registry. Second, the sample size was small. Third, documentation of vaccine recommendations and reasons for lack of vaccination were not universally available due to the retrospective nature of the study. Fourth, data collection did not include live zoster vaccine history and did not include herpes zoster episodes or timing. It was not thought to be necessary to collect data on herpes zoster due to the known high effectiveness of RZV, as well as the known higher risk of herpes zoster in the JAK inhibitor population. Fifth, due to the nature of this study, baseline criteria were only assessed once, meaning that certain herpes zoster risk factors, such as prednisone use, may not be complete.

In conclusion, the majority of patients prescribed JAK inhibitors were not vaccinated with RZV and did not have documentation of vaccine recommendation. Those who were vaccinated were usually ≥50 years of age and timing of vaccine administration did not generally occur in relation to timing of JAK inhibitor initiation, suggesting that JAK inhibitor use did not significantly influence whether or when patients received RZV. Patients who saw a specialty clinic pharmacist were proportionally more likely to receive a recommendation for vaccination.

## Data Availability

Data are available upon request.
